# A Narrative Review of Community-Based Epidemiological Studies on Parkinson’s Disease in India

**DOI:** 10.7759/cureus.80248

**Published:** 2025-03-08

**Authors:** Sarbjeet Khurana, Mandaville Gourie-Devi

**Affiliations:** 1 Epidemiology, Institute of Human Behaviour and Allied Sciences (IHBAS), New Delhi, IND; 2 Neurology, Institute of Human Behaviour and Allied Sciences (IHBAS), New Delhi, IND

**Keywords:** community-based studies, epidemiology, neurodegenerative disease, parkinson’s disease, prevalence rate

## Abstract

Parkinson's disease (PD) is the second most prevalent neurodegenerative condition, following Alzheimer's disease. Worldwide, the prevalence of PD differs among various populations and geographical areas. In our nation, there is a paucity of population-based epidemiological research that can effectively ascertain the prevalence of PD. In this review article, we have attempted to examine the prevalence rates gathered from the neuro-epidemiological studies in India.

We searched for published articles on the prevalence rate of PD in India from PubMed and Google Scholar, using the keywords "prevalence", "burden", "epidemiology", "Parkinson’s disease", and "India". The review includes all the community-based studies that calculated the prevalence rate of PD. There are few community-based studies on the prevalence rate of PD. The observed prevalence rates vary a lot in urban and rural areas; also, urban areas show higher prevalence rates than rural areas. Some studies suggest that Parkinson's disease is more common among males, while others indicate a higher prevalence among females. Almost all research suggests that the prevalence rate increases with age for both males and females. The study carried out at the senior care facility revealed that the elderly had a limited understanding of Parkinson's disease.

Epidemiological studies on PD are crucial to expanding our knowledge about PD. Given the variations in regions, ethnic groups, and cultures, conducting a comprehensive nationwide multi-center study is essential to investigate the prevalence and factors contributing to PD. Initiatives to raise awareness about PD are also the need of the hour.

## Introduction and background

Parkinson’s disease (PD) was first mentioned in Western medicine as a "shaking palsy" by Dr. James Parkinson in 1817 [1]. Notably, references to similar conditions can be found in ancient Ayurvedic and Vedic texts that date back centuries. Vedic literature includes mention of symptoms and treatments related to conditions similar to PD in ancient times. The term “kampavata” originates from the Sanskrit words “kampa” (tremors) and “vata” (a dosha). This condition is characterized by involuntary tremors, stiffness, and challenges in voluntary movements, exhibiting symptoms that closely resemble those of PD [2, 3].

After Alzheimer's disease, PD is identified as the second most common neurodegenerative disorder [4]. Lau and Breteler reported that an estimated 10 million people in the world, representing approximately 0.3% of the world’s population and about 1% of those who are above 60 years of age, are suffering from PD [4]. Parkinson’s disease is a complex of symptoms, both motor symptoms (bradykinesia, slowness of movement, resting tremor, rigidity, and postural instability) and non-motor symptoms (cognitive impairment, depression, anxiety, sleep disturbances, olfactory dysfunction, orthostatic hypotension, and urinary incontinence) [5]. There is a progressive neurodegeneration in PD that mainly affects movement and balance. It is triggered by the depletion of dopamine-producing cells in the brain [6]. The motor as well as non-motor symptoms have the potential to considerably disrupt a person's everyday activities and general well-being. The available literature suggests that men have a higher chance of developing PD compared to women [7].

Worldwide, neurological disorders are becoming the foremost reason for disability [8]. Dorsey et al., in 2016, estimated the global, regional, and national burden of PD and reported that 6.1 million people worldwide had PD. The increase in life expectancy and hence aging is leading to an increase in the burden of neurodegenerative disorders, including PD. The prevalence in India was roughly 10% of the global burden, approximately 5.76 lakhs (0.576 million) [9]. Asia has six of the most populated countries, and the number of PD patients is expected to increase in these countries from 2.7 million to 6.17 million in 2030 [10].

The epidemiology of PD varies across different populations and geographic regions. In comparison to developing countries, its occurrence is more common in developed countries. The prevalence of PD is also influenced by racial differences and ethnicity, which can be conclusively established through future large-scale studies conducted at both national and international levels [11].

We had done this review with the objective of understanding the prevalence rate and hence the burden of PD in India. There is a paucity of community-based epidemiological studies in India, and it is the need of the hour to understand the epidemiology of PD in terms of its prevalence rate. This will enable us to assess the burden of this disease and hence empower the healthcare delivery system and the policymakers to make meaningful strategies catering to the preventive and therapeutic needs for PD in our country.

## Review

Methodology

We conducted the literature search in databases such as PubMed and Google Scholar using keywords such as "prevalence", "burden", "epidemiology", "Parkinson’s disease", and "India". The community-based epidemiological studies that provided the prevalence rate of PD as an output were included in this review.

Results 

Community-Based Studies

There are only a few studies to determine the prevalence of PD in India. We discuss the salient findings of these studies in this article.

Prevalence Rates From Studies Done in Both Urban and Rural Areas

Gourie-Devi et al. carried out a neuro-epidemiological study in the semi-urban and rural regions of Gowribidanur, located near Bangalore, Karnataka, in South India [12]. They conducted a comprehensive house-to-house survey in the town, which had a population of 17,734. In the rural area with a population of 101,556, a systematic random survey was performed in every third house, which provided a sample size of 39,926. In total, the study surveyed a population of 57,660. The research was conducted from March 1982 to June 1984 and comprised three specific stages: a preparatory phase, a study phase, and a verification and treatment phase. The screening tool used in the study was termed the NEPSIG protocol, short for Neuro-epidemiological Study in Gowribidanur [13]. The investigators developed a symptom checklist for screening common neurological disorders, in which items numbers eight and 10 were used for screening PD. The sensitivity and specificity of NEPSIG for common neurological disorders were 95% and 99%, respectively. Out of 57,660 cases examined, there were four cases of PD, resulting in a prevalence rate of seven per 100,000 population. In the semi-urban region, there was only one case of PD, leading to a prevalence rate of six per 100,000, while in the rural area, six cases were identified, yielding a prevalence rate of eight per 100,000 [12].

A pilot survey was carried out [14], followed by a large-scale population-based survey for neurological disorders by Gourie-Devi et al. in 102,557 individuals in urban and rural Bangalore in Southern India [15]. The survey was conducted between January 1^st^, 1993 and September 30^th^, 1995. The WHO questionnaire was used with modifications to detect a wide range of neurological diseases, also called later the NEPSIG or National Institute of Mental Health and Neurosciences (NIMHANS) protocol [12]. The crude prevalence rate for PD was 33 per 100,000, and the age-adjusted prevalence rate was 76 per 100,000. The prevalence rate was higher in the rural area as compared to the urban area (41/100,000 vs. 14/100,000). Authors reported that there were higher rates of neurological disorders among elderly adults in their study, possibly due to cerebrovascular disorders, PD, and dementia [15].

A population-based study was conducted by Gandhi et al. from June 2017 onwards for a period of one year in the urban and rural areas of district Kangra in Himachal Pradesh with the aim to determine the prevalence and pattern of neurological disorders [16]. A total population of 10,000 was screened, 9,000 individuals from rural and 1000 from urban areas; the sample size was determined as per the probability proportional to size. A two-phase design including the screening phase and clinical evaluation was used; the survey was conducted house to house. The NIMHANS protocol was used for screening of the neurological diseases. The total crude prevalence rate of PD was 40 per 100,000, with 200 per 100,000 in urban areas and 22.22 per 100,000 in rural areas. Cases were distributed equally in males and females [16].

Prevalence Rates From Studies Done in Urban Areas

Bharucha et al. conducted a door-to-door population survey on 14,010 people of the Parsi community living in the colonies of Bombay [17]. Medical students and social workers administered a screening questionnaire based on the WHO research protocol for measuring the prevalence of neurological diseases in developing countries (1981) [18] for identifying PD, followed by the clinical examination by the neurologists. The frequency of PD was calculated as the point prevalence rate on March 1, 1985 (prevalence day). A total of 46 people, 25 males and 21 females, were diagnosed as PD cases on the prevalence day. The crude prevalence of PD was reported as 328.3 per 100,000 population. The age-specific prevalence was higher in elderly age groups for both males and females. The age-adjusted rate (AAR) was 192 per 100,000 population and was higher in males (234.8 per 100,000) as compared to females (153.8 per 100,000) [17].

Das et al. conducted a population-based epidemiological study using a stratified random sample from Kolkata to study the prevalence of major neurological disorders, such as epilepsy, stroke, dementia, and parkinsonism [19]. The NIMHANS questionnaire with two added questions for the movement disorders was used in the study for screening of neurological diseases. The study involved a total of 52,377 participants. The AARs per 100,000 population for major neurological disorders were calculated based on the Kolkata urban population 2001, the Indian urban population 2001 census data, and the US population from the year 2000. For PD, the crude prevalence rate was found to be 45.82 per 100,000. The AAR for the Kolkata population in 2001 was 45.82, the Indian urban population in the 2001 census data was 30.87, and the US population in 2000 was 71.64 per 100,000. Sex-specific prevalence was 32.58 among men and 60.60 per 100,000 among women. Age- and sex-specific prevalence increased progressively with age for both genders starting from the fourth decade of life. The highest rates were observed in the eighth decade for women and beyond the eighth decade for men. Sex-adjusted prevalence showed that the disorder was more frequent among women [19].

Das et al. screened a population of 100,802 from Kolkata city between the years 2003 and 2007 by prospective population survey in three stages (in the first stage, a team consisting of trained field workers screened the cases; in the second stage, cases were examined and confirmed by a specialist doctor; and in the third stage, a movement disorders specialist reviewed all surviving cases after one year from the last screening by home visits) [20]. The Family Screening Questionnaire (FSQ) was used in the study for screening neurological diseases. The AARs were calculated by adjusting directly to the World Standard Population. A total of 41 cases of PD were found (18 in men, 23 in women), representing a crude prevalence of 40.67 per 100,000 (33.83 per 100,000 in men and 46.23 per 100,000 in women). The age-specific prevalence rate increased from 1.44 per 100,000 in the age group less than 40 years to 621.11 per 100,000 people in the age group above 80 years of age. In males, the rate increased from 0 in the age group less than 40 years of age to 425.53 per 100,000 in the age group above 80 years of age, whereas in females it was 3.05 in the age group less than 40 years of age and 806.45 per 100,000 in the age group above 80 years of age. Age-adjusted prevalence was 52.85 per 100,000 (45.15 in men and 60.34 per 100,000 in women) [20].

Prevalence Rates From Studies Done in Rural Areas

Razdan et al. conducted a study in the Kuthar Valley in south Kashmir to study the prevalence and pattern of neurological diseases [21]. A house-to-house survey was done in the rural population of 63,645 based on the WHO research protocol (1981) [18]. On the prevalence day, i.e., November 1, 1986, the prevalence of PD was reported as 14.1 per 100,000. The crude prevalence rate was 134 per 100,000 in the age group above 50 years and 247 per 100,000 in persons above 60 years of age [21].

A house-to-house neuro-epidemiological survey was carried out in Malda district [22], 350 km away from Calcutta on a rural population of 37,286 (males: 18,057; females: 19,229) based on the WHO protocol (1981) [18]. The crude prevalence rate of PD was reported as 16 per 100,000. Age-specific disease prevalence showed a progressive increase in rate with advancing age. It was 86.02 per 100,000 in 41 to 50 years of age, 128.07 per 100,000 in 51 to 60 years of age, and 260.30 per 100,000 above 61 years of age. The prevalence rate in males was higher than in females (22.15 per 100,000 versus 10.4 per 100,000) [22].

Saha et al. carried out a study to assess the frequency of neurological disorders among the rural population in eastern India [23], based on the WHO protocol (1981) [18]. From May 1992 to April 1993, a total of 20,842 individuals were screened in two phases over the course of one year. The crude prevalence rate of PD was noted to be 53 per 100,000 people, while the age-standardized prevalence rate was 68 per 100,000. The age-adjusted prevalence was calculated based on the population of the USA in the year 1990 as the standard population. The proportion of females suffering from PD was much higher as compared to males. The age-adjusted prevalence rates in females increased from 214.2 per 100,000 in the age group of 51-60 years to 1666.7 per 100,000 in those above 70 years of age, whereas in males it increased from 164.2 per 100,000 in the age group of 51-60 years to 552.5 per 100,000 in those above 70 years of age [23].

A door-to-door cross-sectional study was done in the rural population of villages of Anand, a district of Gujarat, from 15^th^ September 2019 to 28^th ^February 2020 [24]. This was a three-stage study: two-stage screening and then a clinical examination. A screening tool developed by Sarangmath et al. [25] was used in the study. A total population of 18,896 was screened, and the crude prevalence rate of PD was identified as 42.3 per 100,000 individuals, with a notably higher prevalence of 308.9 per 100,000 in the population aged 60 and above. These data reflect a trend of rising disease prevalence associated with age. The sex-specific prevalence was higher in males, 31.75 per 100,000 population, as compared to 10.58 per 100,000 population in females [24].

Prevalence Rates From Studies Done in Tribal Areas

Mansukhani et al. conducted a community-based cross-sectional study to determine the prevalence of neurological disorders in the tribal area of Kaparada, Gujarat [26]. Surveys were carried out door-to-door in the villages of Moti Vahiyal, Arnai, and Chavshala, located in the Kaparada taluka of Valsad district. The authors used Gourie Devi's adaptation of Schoenberg's two-stage methodology [27] for gathering data from 8,217 people across 1,464 households. The screening questionnaire was created by a group of neurologists and neurophysiotherapists and was translated into Gujarati. All individuals diagnosed with movement disorders were over 40 years old, with 80% of them over 60. The highest prevalence was observed in the 60-69 age group. The crude prevalence rate of PD was 35.51 cases per 100,000 population, with a slight female preponderance [26].

A population-based survey for neurological diseases was conducted in two tribal districts of Kinnaur, Lahaul, and Spiti, and two tribal blocks of district Chamba (Pangi and Bharmour) of the state of Himachal Pradesh, from June 2017 to November 2018 [28]. The sample size of 10,000 was calculated, and study subjects were included by the cluster randomized sampling technique. In the three identified districts, 40 clusters were selected, and 250 subjects were inducted from each cluster. The study was conducted in two phases, a screening phase and a clinical evaluation phase. The modified NIMHANS protocol was used for the screening of neurological diseases. The response rate for phase 2 was 82.55%, and the crude prevalence of parkinsonism was calculated to be 60.57 per 100,000 population. The prevalence was higher in males as compared to females (36.34 versus 24.23 per 100,000 population) [28].

Study in Old Age Homes

A movement disorder neurologist examined 612 elderly residents of old age homes in Bangalore, South India, to study the awareness and occurrence of parkinsonism and possible PD [29]. Parkinsonism was diagnosed in 17.8% (109/612), possible PD in 1.5% (9/612), and definite PD in 16.3% (100/612). It was reported by the author that the knowledge about PD was deficient in these elderly residents even when the occurrence of this disease was high. It was concluded that if their awareness level is improved, their quality of life would definitely improve [29].

A summary of community-based studies on the prevalence of PD in India is presented in Table 1.

**Table 1:** Community based studies mentioning prevalence of Parkinson’s disease in India x: not available/reported/calculated by the author in the article

S. No.	Author	Year of survey	Place	Type of population	Population size	Crude prevalence rate per 100,000	Age‑adjusted rate per 100,000
1.	Gourie‑Devi M et al. [12]	1982‑1983	Gowribidanur, Karnataka	Total	57,660	7	x
				Semiurban	17,734	6	
				Rural	39,926	8	
2.	Bharucha NE et al. [17]	1985	Bombay, Maharashtra	Urban Parsi community	14,010	328	192
3.	Razdan S et al. [21]	1986	Kuthar Valley, Himachal Pradesh	Rural	63,645	14	x
4.	Das and Sanyal [22]	1989‑1990	Malda, West Bengal	Rural	37,286	16	x
5.	Saha SP et al. [23]	1992‑1993	South 24 Parganas, West Bengal	Rural	20,842	53	68
6.	Gourie‑Devi M et al. [15]	1994‑1995	Bangalore, Karnataka	Total	102,557	33	76
				Urban	51,502	14	
				Rural	51,055	41	
7.	Das SK et al. [19]	2003‑2004	Kolkata, West Bengal	Urban	52,377	46	74
8.	Das SK et al. [20]	2003‑2007	Kolkata, West Bengal	Urban	100,802	41	53
9.	Mansukhani KA et al. [26]		Tribal region of Kaparada, Gujarat	Tribal	8,217	35.51	x
10	Gandhi MK et al. [16]	June 2017-June 2018	Kangra district, Himachal Pradesh	Total	10,000	40	x
				Urban	1,000	200	
				Rural	9,000	22	
11	Je G et al. [24]	September 2019-February 2020	Villages of Anand, Gujarat	Rural	18,896	42.3	x
12	Bhardwaj A et al. [28]	June 2017-November 2018	Two tribal districts of Kinnaur, Lahaul, and Spiti, and two tribal blocks of district Chamba (Pangi and Bharmour), Himachal Pradesh	Tribal	10,000	60.6	x

Discussion

There is a paucity of population-based epidemiological studies to accurately deduce the occurrence and prevalence of PD in India. The prevalence studies reported above provide us with some data to ponder. The total crude prevalence rate (both semi-urban/urban and rural) was reported by only three studies [12,15,16]. The study done in the southern part of the country during the 1980s reported a prevalence of seven per 100,000; the second study done in Bangalore in 1994-95 reported a crude prevalence rate of 33 per 100,000, and the study done in northern India nearly two to three decades later reported a prevalence rate of 40 per 100,000 [12,15,16]. This may point towards the rising trend of PD but needs further validation by conducting nationwide multi-centric longitudinal studies.

The prevalence rate from the available studies in the urban area varied from 14 to 328 per 100,000 population [15,16,17,19,20]. The Parsi population is a special population as per ethnicity; their rates cannot be projected as those of the general population, so excluding their rates, the range of urban prevalence varies from 14 to 200 per 100,000 population [15, 16, 19, 20]. In the rural area, the prevalence varied from eight to 53 per 100,000 population [12,15,21-24]. In the tribal areas, only two studies have been done, one in the western part of the country and another in northern India. The prevalence rate was 35.5 and 60.6 per 100,000 population, respectively [26,28]. Almost all the studies have reported a progressive increase in prevalence rate with advancing age. This aligns with research conducted in different regions of India [30,31].

Age-adjusted prevalence was reported by some of the studies and varied from 53 to 192 per 100,000 population [15,17,19,20,23]. Some of the studies have reported that PD is more common in males as compared to females [16,17,22,24,28], whereas others have reported that it is more common in females, and the reason given by some of the authors is the higher life expectancy of females in their study sites [19, 20, 23, 26]. The hospital-based studies of PD in our country have reported that the proportion of males was higher as compared to females, and most of the study patients were in age groups more than 60 years of age [32-34]. The relationship between gender and PD has been examined by researchers worldwide, and it is of value not only in research but also has clinical implications; hence, more studies are needed to conclusively comment on this.

The old age home study pointed out low awareness levels about PD in the elderly [29]. There is a critical need for greater health awareness regarding PD, which can lead to early detection, improved treatment outcomes, and better quality of life for patients. Many people, including patients and caregivers, remain unaware of the early symptoms, such as tremors, rigidity, and slowed movement, leading to delayed medical intervention. Increased awareness can encourage timely medical consultations, lifestyle modifications, and adherence to appropriate treatment.

Community-based surveys to ascertain the prevalence of PD have been done in various regions of our country, i.e., the southern region [12,15], northern region of the country [16, 28], eastern region [19, 20, 22, 23], and western region [18, 24, 26]. As India is a vast country with several regional, ethnic, and cultural variations, it is the need of the hour to plan and conduct the countrywide multi-centric study to look into the prevalence and correlates of PD. This is of utmost importance as healthcare planning and resource allocation depend on the burden and trend of the diseases. It is crucial to determine through a neuro-epidemiological approach the magnitude and pattern of neurological disorders in India to facilitate planning and prioritizing health needs at the local, regional, and national levels of the healthcare delivery system [30,35].

Community-based surveys face various methodological challenges. Parkinson's disease shares several clinical symptoms with other neurological disorders, making differential diagnosis challenging. The main motor symptoms of PD, namely tremors, bradykinesia (slowness of movement), rigidity, and postural instability, are also seen in conditions like multiple system atrophy (MSA), progressive supranuclear palsy (PSP), essential tremor (ET), and dementia with Lewy bodies (DLB). The low awareness of PD in the communities may also limit the participation of the study participants in screening studies. Further, patients often dismiss early, subtle signs (e.g., tremors, rigidity), leading to underdiagnosis during screening.

All the community-based studies of PD have used the screening tools for common neurological disorders of the WHO or NIMHANS protocol for PD, as there was no specific tool for PD. The non-availability of specific tools for a disease can affect the precision and validity of screening and diagnostic output. Misclassification can lead to wrong labeling as diseased (false positives) or missed interventions (false negatives). False positive cases can lead to erroneous prevalence rates and therefore defeat the purpose of large-scale community-based surveys. The lack of standardized methods for data collection and diagnosis can also lead to variability in study results. For population-based surveys, the validity and precision of screening tools are essential for providing accurate, reliable information that can inform public health decisions. These measures ensure that the tools effectively detect the conditions they aim to identify and do so consistently across a large, diverse population. Maintaining high standards for both validity and precision improves the effectiveness of disease prevention, early detection, and resource allocation. Now, there is a tool by Tanner et al. [36] for PD as well as its modified version by Sarangmath et al. [25], which can be used by researchers and nonmedical professionals to authentically screen PD cases.

Since this review was a narrative review that aimed to describe the prevalence of PD, the analytical epidemiological aspects, namely, the risk factors and newer diagnostic markers, etc., were beyond the scope of this review. Future research can focus on various key thrust areas in the field of PD, the currently known (genetic and environmental) as well as the unknown underlying causes, new disease biomarkers for the early diagnosis and monitoring of the disease, new treatment strategies, and multidimensional research towards improving the quality of life of PD patients. Overall, the future of PD research holds promise for improving early detection, advancing treatment options, and ultimately, finding a cure for this debilitating condition. By focusing on these key areas of research, we can continue to make progress in understanding and managing PD. Multi-disciplinary teams and collaborative efforts are essential to address PD research and advance our understanding of this complex neurological disorder.

## Conclusions

The total crude prevalence rate (both semi-urban/urban and rural) of PD was reported by only three studies. The observed prevalence rate in the urban area was higher than in the rural area. The age-adjusted prevalence rates ranged from 53 to 192 per 100,000 people. Although there are several challenges and limitations in planning and conducting research on PD, neuro-epidemiological research remains essential for advancing our understanding of this debilitating neurological condition. Parkinson's disease is an important public health issue that needs special attention not only from clinicians and public health specialists but also from various allied health branches to address diverse issues faced by PD patients. Health awareness about PD is crucial for early diagnosis, effective management, improved quality of life for patients, reduction of stigma, fostering empathy, and encouraging research efforts for better treatments. Continued innovation in research methodologies, data collection techniques, and cross-disciplinary collaboration could be the solution to overcome the obstacles and drive progress in this critical field of study. It is, hence, imperative to focus on the research, preventive measures, health awareness, early diagnosis, the ways to delay the onset of this disease, and finally the management of the disease to address the clinical and other issues related to the disease.
